# Bioremediation of Oil-Contaminated Soil of the Republic of Kazakhstan Using a New Biopreparation

**DOI:** 10.3390/microorganisms11020522

**Published:** 2023-02-18

**Authors:** Tatiana Vyacheslavovna Funtikova, Lenar Imametdinovich Akhmetov, Irina Filippovna Puntus, Pavel Alexeevich Mikhailov, Nurbol Orynbasaruly Appazov, Roza Abdibekovna Narmanova, Andrey Evgenievich Filonov, Inna Petrovna Solyanikova

**Affiliations:** 1 Pushchino Scientific Center for Biological Research of the Russian Academy of Sciences, G.K. Skryabin Institute of Biochemistry and Physiology of Microorganisms of the Russian Academy of Sciences, Prospect Nauki 5, Pushchino 142290, Russia; 2 Department of Ecology and Chemical Technology, Korkyt Ata Kyzylorda University, Aiteke Bi Str. 29A, Kyzylorda 120000, Kazakhstan

**Keywords:** biodegradation, crude oil, microbial consortia, PAHs, alkanes, field trials

## Abstract

A new biopreparation is developed to clean soils from oil pollution in the arid climate of the Republic of Kazakhstan. The biopreparation includes bacterial strains *R. qingshengii* F2-1, *R. qingshengii* F2-2, and *P. alloputida* BS3701. When using the biopreparation in a liquid mineral medium with 15% crude oil, laboratory studies have revealed degradation of 48% n-alkanes and 39% of PAHs after 50 days. The effectiveness of the biopreparation has been demonstrated in field experiments in the soil contaminated with 10% crude oil at the K-Kurylys landfill, Republic of Kazakhstan. During the six-month field experiment, the number of oil degraders reached 10^7^ CFU/g soil, which degraded 70% of crude oil by the end of the experiment.

## 1. Introduction

Oil and petroleum products are the most widespread pollutants in the environment. The development and improvement of bioremediation technologies for areas contaminated by oil hydrocarbons are currently an area of active basic and applied research. In many cases, the process of oil degradation can be activated by applying mineral and/or organic fertilizers (biostimulation), as well as by introducing special cultures of microorganisms capable of degrading various oil hydrocarbons (bioaggregation). Stimulation of native microflora is better positioned as it activates a large number of different groups and species of microorganisms. This approach is most justified in areas where there is a regular release of crude oil and oil products into the environment. For example, in areas where there are natural leaks of crude oil from oil fields or at oil production sites. The experts state that about 47% of crude oil releases into the marine environment from natural seeps and 53% are the result of leaks and spills during the extraction process, transportation, refining, storage, and use of oil. This amount of natural crude oil seepage is estimated to be approximately 600,000 metric tons per year (with a possible increase of 200,000 metric tons) [1]. In this case, the natural inhabitants possess oil degradation genes from the exchange by genetic determinants. The situation is much more complex in the case of accidental oil spills, especially if the pollution is extremely large, as were the cases in the USA, Exxon Valdez shipwreck in 1989, Petroleum Deepwater Horizon emergency in 2010 [2], and in China in Dalian in July 2010 [3]. When large quantities of crude oil are introduced into an environment whose natural inhabitants are poorly adapted to deal with this pollution, the introduction of oil-oxidizing microorganisms is not only justified, but also absolutely necessary [4].

Kazakhstan currently ranks in the top 10 countries in oil and gas reserves [5] and has 3% of the world’s total oil reserves. A distinctive feature of oil pollution in the Aral Sea region of Kazakhstan is that these areas are mainly located in a zone with a sharply continental climate and prevailing high temperatures in the summer, and the soil is a salt marsh with 3–4% salt content with very low moisture and organic matter content. The high air temperatures and consequent soil temperatures in the spring and summer in arid hot regions mean that the light and medium oil fractions evaporate in the first days to weeks after an oil spill, leaving the heavy compounds in the soil with a complex structure.

Due to the complex multicomponent composition of crude oil, which varies greatly depending on the field, sharp differences in chemical properties between oil and oil products and the varying natural, climatic, and hydrothermal conditions of oil and oil product’s production, processing, and storage, it is impossible to create a single universal biopreparation oil destructor. Therefore, work on the development of biopreparations to clean the environment following oil pollution and the development of technologies for their application will continue to be relevant.

The aim of this work was to create a consortium of active microbial oil hydrocarbon-degrading organisms as a basis for biopreparation for the bioremediation of oil-contaminated soils in sharply continental climates and determine its effectiveness in both laboratory and field conditions.

## 2. Materials and Methods of Research

### 2.1. Experimental Work Plan

(a) Create of several consortia from the most effective strains of oil destructors from our collection since no active oil-oxidizing strains failed to be isolated from soil samples from the K-Kurylys oil deposit. Study of strains for the presence of degradative plasmids.
(b) Compare the degradative properties of the developed consortia: evaluate the degree of degradation of oil and individual fractions of oil hydrocarbons.
(c) Produce the biopreparation.
(d) Field tests of the new biopreparation. Assessment of the degree of oil degradation under field conditions.

### 2.2. Bacterial Strains

Nine strains of oil and diesel fuel degraders isolated from oil-contaminated soils [6], namely seven rhodococci, *Pseudomonas putida* strain BS3701 (VKM B-2380 D) from the Laboratory of Plasmid Biology, IBPM RAS [7] and *Acinetobacter baumannii* strain 7 from the JSC ‘Biooil’ collection [8], were used in this work (Table 1).

Seven rhodococci strains used in this work were isolated from different samples of oil-contaminated soils [6]. According to the results of the analysis of the nucleotide sequences of 16S rRNA gene fragments, these strains were assigned to the genus Rhodococcus. The nucleotide sequences for the mentioned genes of the investigated bacteria were deposited in GenBank under accession numbers MF359738 (*Rhodococcus* sp. F2-2), MF359739 (*Rhodococcus* sp. T3-4), MF359740 (*Rhodococcus* sp. K1), MF359741 (*Rhodococcus* sp. T3), MF359742 (*Rhodococcus* sp. K2), MF359743 (*Rhodococcus* sp. B1), and MF359744 (*Rhodococcus* sp. F2-1). For these rhodococci, establishing the species affiliation using only 16S rRNA gene sequencing data appears not to be possible since the cut-off areas show 99% similarity to those of several species.

The minimal Evans medium was used with the following composition (g/L or mL/L deionized water) [14]: 50 mM K_2_HPO_4_; 5 M NH_4_Cl—1 mL; 1 M Na_2_SO_4_—1 mL; 62 mM MgCl_2_—1 mL; 1 mM CaCl_2_—1 mL; 0.05 mM (NH_4_)_6_Mo_7_O_24_·4H_2_O—1 mL; and trace elements—1 mL (content: ZnO—0.41 g/L; FeCl_3_·6 H_2_O—5.4 g/L; MnCl_2_·4H_2_O—2 g/L; CuCl_2_·2H_2_O—0.17 g/L; CoCl_2_·6H_2_O—0.48 g/L; H_3_BO_3_—0.06 g/L).

The rich medium used was L-broth [15], containing (%): bacto-tryptone (Pronadisa, Madrid, Spain)—1.0; yeast extract (Pronadisa, Spain)—0.5; and NaCl—1.0.

To obtain an agarized medium, 20 g of agar (Pronadisa, Madrid, Spain) was added. The media were sterilized by autoclaving for 30 min at 1 atmosphere. The oil used in the work was from the Ashisai field (Republic of Kazakhstan) with the following composition: 65.3% unbranched alkanes, 12.4% branched alkanes, 6.7% naphthenes, 3.7% arenes, and 11.9% other compounds.

### 2.3. Cultivation Conditions

Cultivation was carried out in Erlenmeyer flasks with 100 mL of minimal Evans medium supplemented with oil to a final concentration of 2% (*w*/*v*). The inoculating concentration of microorganisms in the medium was 2–3 × 10^6^ cells/mL. After inoculation, flasks were placed on an orbital shaker (120 rpm) and the microorganisms were grown for 10 days at 24 °C.

### 2.4. Visualisation of Plasmids Using Pulse Electrophoresis (PFGE)

The strains were grown with a logarithmic growth phase. The cells were separated by centrifugation and washed in the following solution: 25 mM EDTA and 2.5 M NaCl. The cells were then precipitated and subjected to a freezing procedure at −70 °C and a thawing procedure at +37 °C for 15 min each for better cell lysis. The cells were then resuspended in 0.5 mL of washing solution to a value 2 of OD_620_. To obtain agarose blocks, 0.5 mL of 2% light-melting agarose, melted and cooled to +37 °C, was added to a suspension of cells heated to +37 °C. The cell suspension in agarose was poured into molds and cooled until the gel solidified. These block inserts were then treated sequentially in 5 mL of solutions No. 1 and No. 2 for 24 h each. Lysing solution No. 1 contained 6 mM Tris-HCl (pH 7.5), 1 M NaCl, 100 mM EDTA (pH 8.0), 0.5% Brij—58, 0.2% Deoxycholate, 0.5% Lauroyl sarcosine, and lysozyme 2 mg/mL. Lysis solution No. 2 contained 0.5 M EDTA pH 9–9.5, and 1% Lauroyl sarcosine.

Immediately before use, proteinase K was added to a concentration of 1 mg/mL. Cells were lysed at +37 °C in solution No. 1 and then in solution No. 2 at +55 °C on a shaker. The insertion was washed twice with deionized water for 15 min each and then stored in 1 mL of TE buffer at 10 °C. The separation of high molecular weight DNA fragments was performed by pulse electrophoresis in a Pharmacia-LKB device under conditions recommended by the manufacturer: hexagonal electrode array (120°), 180 V, 14 °C, pulse time was 30 to 70 s over 24 h.

### 2.5. Determination of the Degree of Oil Degradation by Gravimetric Method

Residual oil was extracted from the culture medium with 50 mL chloroform (2:1 *v*/*v*); the extract was separated by centrifugation for 30 min at 4500 rpm and dried by stirring over 3 g anhydrous sodium sulphate. To remove the chloroform, the tare tubes with 5 mL of the extract were incubated at 70–75 °C for 4 h and then at 35–40 °C overnight and weighed.

The fractional composition of residual oil was assessed by adsorption chromatography using micro-columns filled with 0.7 g silica gel 60, 63–200 microns RE (70–230 mesh) (Panreac, Spain). The sorbent was equilibrated with hexane. Samples (50 mg each) obtained by extraction during the weight analysis were dispersed in 1 mL of hexane, applied on microcolumns and left for 20 min. Elution of hydrocarbons was carried out with hexane, benzene, and a benzene/ethanol mixture in a ratio of 1:1 (*v*/*v*) in four portions of 1 mL into tare tubes. The obtained hexane, benzene and alcohol–benzene fractions were evaporated in air and dried in a ventilated desiccator at 75 °C to constant weight. The content of the fractions was calculated in terms of the original sample weight. Losses of the original sample due to irreversible sorption on silica gel in these conditions were conventionally taken as asphaltenes and high molecular weight resinous substances.

### 2.6. Determination of n-Alkanes in Residual Oil by Capillary Gas–Liquid Chromatography

Biopreparations were cultured in Erlenmeyer flasks in Evans mineral medium in the presence of 15% oil at 24 °C for 50 days. The oil was then extracted from the liquid phase with chloroform. The chloroform extract was dried with anhydrous sodium sulphate and evaporated to dryness at room temperature. The polar fraction was purified on aluminum oxide (Al_2_O_3_). Analysis of samples was carried out on Agilent 6890 gas chromatograph with a flame ionization detector, GC column DB-1MS, length 30 m, diameter 0.25 mm, phase thickness 0.25 μm. A standard mixture of n-alkanes (C_11_H_24_-C_36_H_74_) containing pristane and phytane was used for calibration against individual n-alkanes.

### 2.7. Determination of PAH Content by Capillary Gas–Liquid Chromatography

The biopreparations were cultured in Erlenmeyer flasks in Evans mineral medium in the presence of 15% oil at 24 °C for 50 days. The oil was then extracted from the liquid phase with chloroform. The chloroform extract was dried over anhydrous sodium sulphate and evaporated to dryness state. The purification of the chloroform extract for the determination of PAHs was carried out on a Diapac C cartridge (Diapac LLC, Moscow, Russia). The PAH fraction was analysed on an Agilent 1200 liquid chromatograph with a fluorometric detector after transferring acetonitrile heated to 60 °C into a chromatographic vial (Diasfer 110-C18 column (IC Econova LLC, Novosibirsk, Russia), length 250 mm, diameter 4 mm, particle size 5 μm). A standard mixture of PAHs in acetonitrile (thirteen PAHs) was used for calibration against individual PAHs. The concentrations were calculated taking into account the calibration dependence of the peak areas of individual PAHs on their concentration in the standard solution. Calibration curve construction and calculation of PAH concentrations in samples were performed using Chemstation software 4.03.016.

### 2.8. Preparation of a Test Biopreparation Sample

*P. putida* BS3701 and two strains of *Rhodococcus* (F2-1 and F2-2) were cultured in a 10 L fermenter ANKUM-2M (IBI RAS, Pushchino, Russia) with a filling ratio of 0.6. The inoculum for all fermentations was grown on LB medium for 24 h.

A semisynthetic medium of the following composition was chosen for culturing *P. putida* BS3701 strain in the ANKUM-2M fermenter: acid casein hydrolysate, 10 g/L; yeast autolysate, 70 mL/L; (NH_4_)_2_SO_4_—6 g/L; K_2_HPO_4_—2 g/L; glucose—20 g/L; MgSO_4_—0.3 g/L; MnSO_4_, 0.05 g/L; defoamer Sofexil-152» (20% polymethyldisiloxane water-oil emulsion, Sofex-Silicone, Moscow, Russia), 1 mL/L medium; and tap water to 6 L. The cultivation mode: temperature 28 °C; shaker speed 450 rpm; pH 6.8 ± 0.2 (automatically maintained by adding 12% ammonia solution to the medium); and aeration with air 3 L/min from the beginning to 4 h of growth, then 6 L/min until the end of the process.

Cultivation conditions for microorganisms of *Rhodococcus* strains were chosen similar to those for pseudomonads. The composition of the medium differed in the content of acid casein hydrolysate—5 g/L; yeast autolysate—100 mL/L; and peptone—5 g/L. In the middle of the exponential growth phase, diesel fuel was added (1 mL/L).

The cultivation mode was similar to that for *P. putida* BS3701, except for medium acidity (pH 7.0 ± 0.2) and aeration (3 L/min from 0 to 8 h of growth, then 6.0 L/min).

Concentrated microbial suspension was obtained by centrifugation for 30 min on a K70 centrifuge (Janetzki, Poland) at 5000 rpm at 4 °C.

Before lyophilization, the concentrated suspension mixed with protective medium was incubated at −20 °C for 24 h. The protective media used were: for *Rhodococcus*, solution No. 1 (4% thiourea, 8% sucrose, 4% polyglucin, water); and for *Pseudomonas*, solution No. 2 (20% sucrose, water). The ratio of biomass/protective medium was 1:1 by mass. Lyophilization was carried out on a lyophilic drying unit KS30 (Frigera, Kolin, Czech Republic) at 35 °C and a pressure of 0.5 Pa. The obtained dry preparation, with a microbial population of 8.0 × 10^10^ CFU/g, was stored at room temperature in sealed glass vials.

### 2.9. The Field Trial

Field test was conducted on oil-contaminated soil (10%) of K-Kurylys oil-waste landfill (Kyzylorda oblast, RK). Plot No. 1 with area of 4 m^2^ without biopreparation treatment was a control for abiotic degradation of oil hydrocarbons and biodegradation under the action of indigenous microorganisms. The control plot was watered with 10 liters of fresh water. Plot No. 2 of 4 m^2^ was treated with a biopreparation. The concentration of the applied bacteria was 10^7^ CFU/g of soil. The bacteria were applied to the plot in 10 liters of fresh water. In addition, cow manure as organic fertilizer (5 kg/m^2^) and the mineral fertilizer Nitroammophoska (‘Fasko+’ Ltd., Khimki, Russia) (10 g/m^2^) as biogenic sources of nitrogen, phosphorus, and potassium were applied to both plots. The plots were then ploughed over.

To determine the total microbial number and oil content, averaged samples (1 g and 5 g, respectively) were taken from 3–4 different soil areas every 2 weeks. Samples weighing 1 g were resuspended in 9 mL of phosphate buffer and stirred on a Paramix 2 mixer (Juccheim Labortechnik KG, Seelbach, Germany) for 1 min at room temperature and, after serial dilutions, plated onto dishes with agar rich medium and minimal Evans supplemented with diesel fuel. The plates were incubated at 24 °C for 3–7 days. The number of colony-forming units was calculated per 1 g of dry soil.

### 2.10. Statistical Processing of the Results

The results were processed using a built-in statistical dispersion analysis package on Excel (MS Office 2007).

## 3. Results and Discussion

### 3.1. Making Microbial Consortia

There are currently no clear criteria for establishing microbial consortia to produce biopreparations. Most researchers, when developing such consortia, rely on the compatibility of strains and their catabolic activity and the degree of total degradation of oil hydrocarbons as the main parameter for which the consortium is formed. Different approaches can be used to form an consortium of petroleum hydrocarbon-degrading strains: (1) Based on the analysis and subsequent combination of physiological, metabolic, and catabolic traits of microorganisms; and (2) selection by periodic cultivation of a mixed association of chosen microorganisms in a liquid mineral medium with oil as a sole source of carbon and energy [16].

Crude oil is a mixture of hundreds of chemicals. Therefore, microbial consortia consisting of two or more strains have recently been used in bioremediation, as the introduction of a monoculture of hydrocarbon-oxidizing microorganisms into an oil-contaminated environment cannot fully solve the clean-up problem. Polybacterial preparations composed of strains of different taxa, which differ in growth rate, spectrum of consumed substrates, and metabolic features have better adaptation and ecological possibilities [7].

The ability to assimilate oil hydrocarbons is inherent in microorganisms represented by different taxonomic groups [17]. The most common in oil polluted habitats are bacteria from the genera *Rhodococcus*, *Arthrobacter*, *Pseudomonas*, and *Acinetobacter* [18]. Due to their ability to degrade a variety of ecotoxicants, *Pseudomonas* and *Acinetobacter* bacteria are actively studied by scientists around the world in order to apply them for the environmental treatment of various pollutants [19,20,21,22,23].

In chronically contaminated soils, rhodococci are often the dominant group. Having unique biological properties and characterized by wide catabolic capabilities and unique enzyme systems, *Rhodococcus* can degrade chemically diverse xenobiotics [24,25]. These features in combination with the ability to survive under unfavorable environmental conditions make *Rhodococcus* genus representatives promising in the development of preparations for the bioremediation of hydrocarbon-contaminated objects [26,27,28,29].

In the first stage of the work, we had to choose the most effective oil-degrading strains from the available ones in order to create the microbial consortia that degrade hydrocarbons in a wide temperature range. We have previously investigated the physiological, biochemical, and metabolic properties of the petroleum-degrading microorganisms (Table 1). These properties have been described in an article [6].

Strains referenced in Table 1 degrade petroleum hydrocarbons in a wide temperature (4–37 °C) and pH-range (4–9), are resistant to salts (up to 7%), and are able to produce biosurfactants. Bioemulsifier production is known to be an important property of bacteria that increases the bioavailability of insoluble hydrocarbons [30,31,32]. The inclusion of biosurfactant-producing *Rhodococcus* in biopreparations used for the treatment of oil-contaminated environments is feasible.

We examined these strains (Table 1) for the presence of plasmids since conjugative catabolic plasmids also enhance the degradation potential and adaptive properties of rhodococci [33,34]. Horizontal transfer is a key factor in the emergence of new catabolic pathways and the provision of additional carbon and energy sources for rhodococci. The transfer of genes encoding for halogenalkanes, alkenes, biphenyls, and naphthalene catabolism between different *Rhodococcus* strains has been described recently [33,34,35].

**Figure 1:**
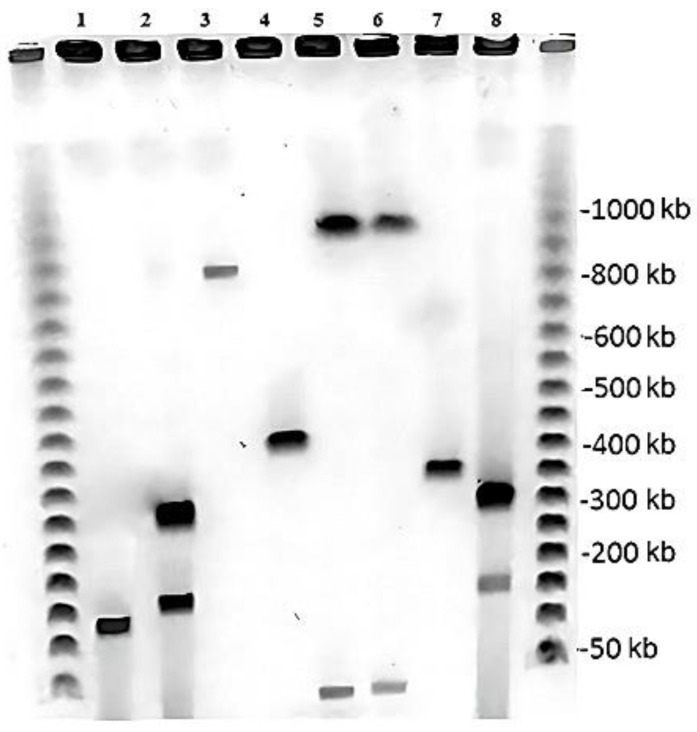
Plasmid profile of the studied *Rhodococcus* degraders. Ladder—λ-phage (BioRad); 1—K3; 2—F2-2; 3—T3-4; 4—K1; 5—T3; 6—K2; 7—B1; and 8—F2-1.

Plasmid DNA isolation has revealed that seven of the effective *Rhodococcus* strains studied carry plasmids ranging in size from 20 to 1000 bp, and two strains carry two plasmids of 150 and 300 bp (Figure 1). BS3701 has previously been shown to contain two plasmids of 56 and 100 bp [9]. *A. baumannii* 7 cells carry small plasmids (6, 12, 20, 42, and 50 kb) [8].

Taking all the above into account, four microbial consortia were composed (Table 2): two include only microorganisms from the genus *Rhodococcus* (consortium No. 1: strains F2-1 and F2-2; consortium No. 2: strains F2-1, F2-2, and T3-4), and one consortium was supplemented with an effective PAH-degrading microorganism from the genus *Pseudomonas* (consortium No. 3: strains F2-1, F2-2, and BS3701). Consortium No. 4 (strains F2-1, F2-2, and 7) was enhanced with an effective short-chain n-alkane degrader from the genus *Acinetobacter*.

### 3.2. Study of Degradative Activity by Composed Microbial Consortia

In the second stage of the study, the microbial consortia were cultured in Erlenmeyer flasks at 24 °C in liquid mineral Evans medium containing 2% oil for 10 days to analyze the degradation process. The initial concentration of microorganisms in the medium was 2–3 × 10^6^ CFU/mL. At the end of the experiment, the number of microorganisms increased to 10^8^–10^9^ CFU/mL. The residual concentration of oil hydrocarbons after culturing was estimated by a gravimetric method (Table S1). Abiotic degradation was assessed by oil loss in flasks without the addition of microorganisms.

**Figure 2:**
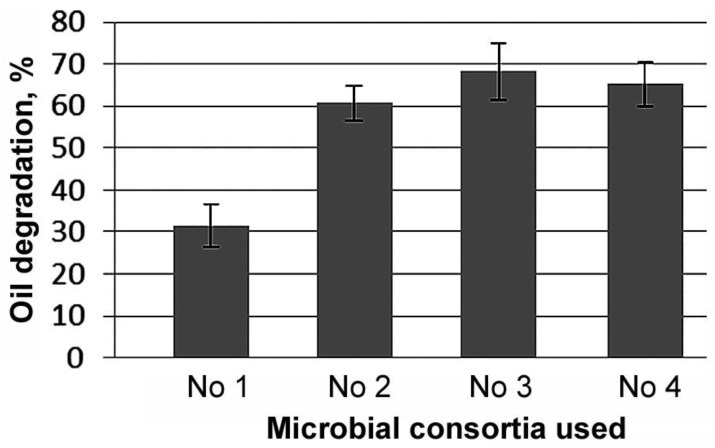
Degradation of oil by different microbial consortia when cultured in Evans liquid mineral medium containing 2% oil for 10 days at 24 °C (minus abiotic degradation); abiotic oil loss was 19 ± 5%. Bars—standard deviation.

After 10 days of cultivation at 24 °C in medium without the microorganisms, the oil loss was 19% due to evaporation. The oil removal in the medium with the microorganisms of consortium No. 1 (two *Rhodococcus* strains) was 50%. With the addition of another *Rhodococcus* strain (consortium No. 2), oil removal was 80%, and with the addition of the *Pseudomonas* strain (consortium No. 3) and *Acinetobacter* strain (consortium No. 4), oil removal was 87 and 84%, respectively (Figure 2). After subtracting abiotic degradation, the degree of oil biodegradation by the different consortia ranged from 30 to 70%.

Thus, when a third strain was added to consortium No.1, consisting of two strains of the genus *Rhodococcus*, the oil degradation efficiency of consortia No. 2–4 was increased by a factor of 2, amounting to 61 to 68%.

The use of fractionation allows residual oil to be separated into three conditional fractions: hexane fraction, contains paraffin–naphthenic and aromatic hydrocarbons; benzene fraction, contains polycyclic aromatic hydrocarbons; alcohol–benzene fraction contains naphthenic acids and alcohol–benzene resins (Table 3). Some residual oil remains bound to silica gel after elution of the hydrocarbons with solvents. This fraction represents asphaltenes and high molecular weight resinous substances.

Samples (50 mg) from the weight analysis were dispersed in hexane and applied to silica gel microcolumns. The weight of the residual oil sample (50 mg) applied to the column was taken as 100% for fractional calculations. The fraction content was calculated as a percentage based on its weight in relation to the control.

As shown in Table 4, in the control (oil without microorganisms), the hexane fraction was 48%, the benzene fraction 18%, and the ethanol–benzene fraction 10%. In the control, up to 24% of the applied oil was sorbed on the column.

To illustrate the consumption of individual oil fractions, the loss of a particular fraction was calculated as a percentage of the control sample. The weight of the fraction in the control sample (e.g., hexane) was 24 mg (Table 3). This was taken as 100%.

All consortia utilized the hexane fraction of oil most efficiently, i.e., they mainly consumed paraffin–naphthenic and monoaromatic hydrocarbons (hexane fraction) as a carbon and energy source (Table S2). The loss of this fraction ranged from 21 to 37%. The benzene fraction compounds (polycyclic aromatic hydrocarbons) were also effectively consumed, the loss being 22–56%.

The ethanol–benzene fraction in residual oil samples under study increased due to the accumulation of degradation products and an enlarged proportion of asphaltenes and high molecular weight resinous substances in the residual oil samples after the degradation by microbial consortia.

Consortium No. 3, consisting of a strain of the genus *Pseudomonas* (BS3701) and two strains of the genus *Rhodococcus* (F2-1, F2-2), was the most efficient because the loss of hexane and benzene fractions was the highest, 37% and 56%, respectively. This consortium efficiently consumed both paraffin–naphthenic and polycyclic aromatic hydrocarbons. The addition of strain *P. alloputida* BS3701 to two strains of the genus *Rhodococcus* (F2-1 and F2-2) (consortium No. 3) resulted in twice the loss of the hexane fraction and three times the loss of the benzene fraction compared to consortium No. 1, consisting of two *Rhodococcus*.

To select a more efficient consortium, the consumption of oil alkanes and PAHs by microbial consortia No. 3 and No. 4 was evaluated by culturing them in Erlenmeyer flasks in Evans mineral medium in the presence of 15% oil at 24 °C for 50 days. The content of alkanes and PAHs in the residual oil was determined by capillary gas–liquid chromatography (Figures S1 and S2). Consortium No. 3 (F2-1, F2-2, BS3701) was shown to degrade 48% of the tested n-alkanes compared to the control (medium with oil without microorganisms). For consortium No. 4, this value was 27%. Consumption of individual oil alkanes by microbial consortia is shown in Table S2.

Consortium No. 3 degraded 39% of polycyclic hydrocarbons (number of rings 3–6) as compared to control. For consortium No. 4, the value was 23.6%. Consumption of individual PAHs by microbial consortia is presented in Table S3.

The use of strains of the genus *Pseudomonas* to clean PAH-contaminated soil is an effective bioremediation technique [36,37,38]. As shown in Table S3, consortium No. 3, which includes *P. alloputida* BS3701, is more efficient in PAH degradation (39%) compared to consortium No. 4 (24%) and is chosen to carry out further research in the open oil-contaminated environment.

### 3.3. Field Trials of Biopreparation Based on Consortium No. 3 (R. qingshengii F2-1, R. qingshengii F2-2, P. alloputida BS3701)

A total of 62% of the Republic of Kazakhstan is occupied by oil and gas areas, and there are 172 oil fields, of which more than 80 are under development. More than 90% of oil reserves are concentrated in the 15 largest oil fields—Tengiz, Kashagan, Karachaganak, Uzen, Zhetybai, Zhanazhol, Kalamkas, Kenkiyak, Karazhanbas, Kumkol, North Buzachi, Alibekmola, Central and Eastern Prorva, Kenbai, and Korolevskoye. Oil fields can be found in six of the fourteen provinces of Kazakhstan. They are the Aktobe, Atyrau, West Kazakhstan, Karaganda, Kyzylorda, and Mangystau provinces [39].

Bioremediation based on the principle of activation of soil microflora is not sufficiently effective in high-temperature conditions of the Aral Sea region. Several biopreparations to combat oil contamination of soil and water have been developed in Kazakhstan (Table 5).

**Table 5:** Patented biopreparations applied for bioremediation of crude-oil-spilled soils in Kazakhstan.

Title	Microorganisms	Reference
Consortium	*Acinetobacter calcoaceticum* 2A *Microbacterium lacticum* 41-3	[40] [41]
‘Kazbiorem’	*Rhodococcus erythreus* AT7 *Dietzia maris* 22K	[42]
’Bakoil-KZ’	*Rhodococcus erythropolis* 7A *Micrococcus roseus* 40 *M. roseus* 34	[43] [44]
‘Peroil’	*Rhodococcus erythropolis* DP 304 *Micrococcus luteus* B1Ag8G	[45] [46]
algae-bacterial consortium	*Phormidium* sp. K11 *Pseudomonas* sp. N2 *P. stutzeri* A1 *P. alcaligenes* A5	[47]

Only the results of laboratory tests of the biopreparation “Kazbiorem” (two actinobacteria—*Rhodococcus erythreus* AT7 and *Dietzia maris* 22K) in the oil-polluted soil of Zhanatalap (5.3% pollution) and Kalamkas (2.4% pollution) fields of Western Kazakhstan are known [42]. The degree of destruction in the soil from Zhanatalap on the 7th day reached 88,23% and in the soil from Kalamkas, 97,4% (in control 9.3 and 6.4%, respectively). A consortium of *Acinetobacter calcoaceticum* 2A and *Microbacterium lacticum* 41-3 strains in the model experiments reduced the oil concentration (initially 10%) by 64.4% in three months [48].

Field experiments on ‘Khimpromservice Actobe’ LLC oil sludge landfill (Zhanazhol field) using cyanobacterial association were performed [47]. To obtain the biopreparation, cyanobacterial paste was first obtained for a month and then *P. stutzeri* A1, *Pseudomonas* sp. N2, and *P. alcaligenes* A5 bacteria were introduced into it. In 6 months (April to October 2011) with periodic wetting after the introduction of oil-oxidizing microorganisms, the concentration of C_14_–C_27_ and C_32_–C_34_ alkanes decreased 3–10 times in the soil; C_12_-C_13_, C_28_-C_31_ decreased below the threshold of instrument sensitivity.

“Bakoil KZ”, containing four actinobacterial strains, was applied at the West Dala LLP test site [43]. The natural process of self-purification of the soil from oil under favorable conditions led to oil destruction by 18% after one month. After application of “Bakoil-KZ”, the cleaning process increased by 80% minus control. Idrisova et al. [44] (2015 patent 30176) performed field trials at the ‘K-Kurylys’ landfill of Kumkol field in Kyzylorda oblast using different combinations of nitroammophos, biovermicompost, zeolite, and biopreparation “Bakoil-KZ”. After two months in control, the loss of oil in the soil was 18.3%, whereas the oil utilization when using the biopreparation and ameliorants was 68.3–82.2%.

The biopreparation “Peroil” consists of two strains of actinobacteria [45]. Sites with a total area of 3 hectares subjected to regular and single spills of specific oil products were selected to test “Peroil”: oil (0.3–0.5%), oil (0.7–1.0%), diesel fuel (2.0–2.5%), fuel oil (1.0–2.3%), fuel oil (over 10%), and sludge-like wastes (9.8–14.0%). During 1.5 months of remediation, a 10-fold reduction in petroleum product concentration was observed at all investigated sites. Myrkhalykov et al. [46] used the sorbent bentonite as an immobilizer for ‘Peroil’ diluted in water. Two soil plots contaminated with 5.4% oil and 4.7% fuel oil were treated by applying bentonite with “Peroil”. In 15 days we observed a 66.2% decrease in oil concentration in the study area and in 20 days a 73.1% decrease in fuel oil concentration.

We tested the developed consortium in May–July 2021, on the territory of K-Kurylys oil waste landfill (Kyzylorda Region). It should be noted that the ambient temperature during the field experiment was 15–45 °C during this period. The biopreparation was dissolved in fresh water and applied at the rate of 1.2 × 10^8^ CFU/g of soil. An oil-contaminated area without biopreparation treatment served as a control. As the soil was characterized by very low moisture and organic matter content, additional organic and mineral fertilizers were applied to both plots. The soil was then loosened and periodically watered.

Studies of the number of indigenous microorganisms at the beginning of the experiment in the oil-contaminated soil of the K-Kurylys oil waste dump showed that the total number of microorganisms was 3.2 × 10^6^–4.0 × 10^6^ CFU/g, and the number of number of microorganisms-destructors did not exceed 1% (Table 6). When we applied the biopreparation to the soil, the number of microorganisms at the beginning of the experiment was 1.2 × 10^8^ CFU/g of soil and 80% of which were microorganism destructors.

**Figure 3:**
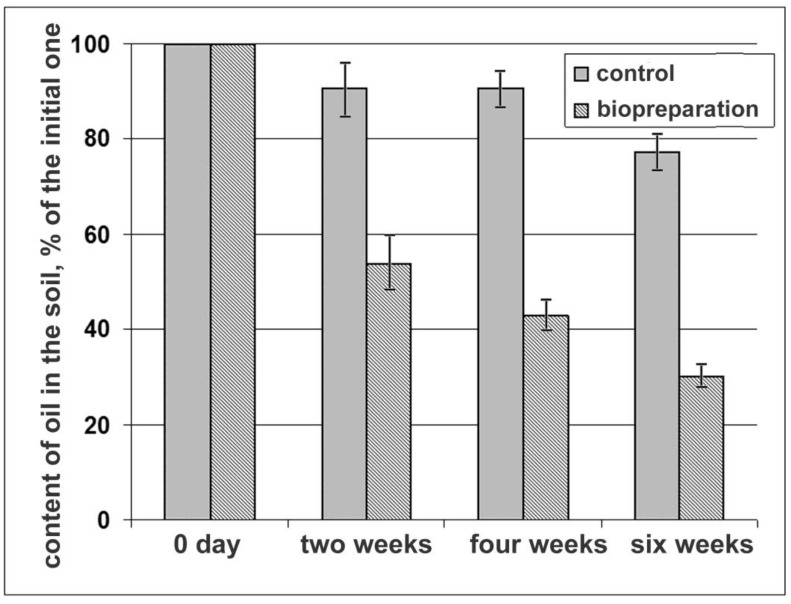
Oil removal in the soil in the control plot and the biopreparation-treated plot (bars—standard deviation).

By the end of the six-month field experiment, the number of oil destructors was 10^7^ CFU/g of soil, which ensured the degradation of 70% of oil (Figure 3). In the control, the oil degradation was 23%.

Thus, the microbial consortium obtained by us on the basis of strains *R. qingshengii* F2-1, *R. qingshengii* F2-2, and *P. alloputida* BS3701 successfully proved itself in field experiments on the contaminated soils of K-Kurylys oil-waste landfill. The proven technology of production and application of this consortium allows it to be considered as a basis for a new effective biopreparation.

## Figures and Tables

**Table 1:** Characterization of strains used in the work.

Strain	Phenotype	Source
*Rhodococcus qingshengii* F2-1	Npht^+^ Dsf^+^ Hde^+^ Phe^+^ Bnz^+^	[6]
*R. qingshengii* F2-2	Npht^+^ Dsf^+^ Hde^+^ Phe^+^	[6]
*Rhodococcus* sp. T3	Npht^+^ Dsf^+^ Hde^+^ Phe^+^ Bnz^+^ Ebnz^+^	[6]
*Rhodococcus* sp. B-1	Npht^+^ Dsf^+^ Hde^+^ Phe^+^ Ebnz^+^	[6]
*Rhodococcus* sp. T3-4	Npht^+^ Dsf^+^ Hde^+^ Phe^+^ Bnz^+^ Ebnz^+^	[6]
*Rhodococcus* sp. K2	Npht^+^ Dsf^+^ Hde^+^ Phe^+^	[6]
*Rhodococcus* sp. K1	Npht^+^ Dsf^+^ Hde^+^ Phe^+^ Bnz^+^ Ebnz^+^	[6]
*P. alloputida* BS3701	Npht^+^ Dsf^+^ Nah^+^ Phn^+^ Sal^+^ Gnt^+^	[7]
*A. baumannii* 7	Npht^+^Dsf^+^Hde^+^ Oct^+^ Dec^+^ Non^+^	[8]

Note. Ability to grow using: Npht^+^—crude oil; Dsf^+—^diesel fuel; Hde^+^—hexadecane; Phe^+^—phenol; Bnz^+^—benzene; Ebnz^+^—ethylbenzene; Oct^+^—octane; Dec^+—^decane; Non^+^—nonane; Nah^+^—naphthalene; Phn^+^—phenanthrene; Sal^+^—salycilate; Gnt^+—^gentisate. *Pseudomonas alloputida* strain BS3701 contains two plasmids (pBS1141 and pBS 1142). Plasmid pBS1141 carries naphthalene biodegradation genes [9]. *R. qingshengii* F2-2 contains plasmids harboring genes of *n*-alkane biodegradation [10,11,12,13].

**Table 2:** Composition of oil-degrading microbial consortia.

Consortium	Strains
No. 1	*R. qingshengii* F2-1 *R. qingshengii* F2-2
No. 2	*R. qingshengii* F2-1 *R.qingshengii* F2-2 *Rhodococcus* sp. T3-4
No. 3	*R. qingshengii* F2-1 *R. qingshengii* F2-2 *P. alloputida* BS3701
No. 4	*R. qingshengii* F2-1 *R. qingshengii* F2-2 *A. baumannii* 7

**Table 3:** Fractional analysis of oil after its degradation by different microbial consortia for 10 days at 24 °C (average value ± standard deviation).

Fraction	Weight, mg, and Percentage				
	Control	Consortium No. 1	Consortium No. 2	Consortium No. 3	Consortium No. 4
Hexane	24 ± 1 **48%**	19 ± 2 **38%**	16 ± 1 **32%**	15 ± 1 **30%**	16 ± 1 **32%**
Benzene	9 ± 2 **18%**	7 ± 1 **14%**	7 ± 1 **14%**	4 ± 1 **8%**	7 ± 1 **14%**
Alcohol–benzene	5 ± 1 **10%**	8 ± 1 **16%**	7 ± 1 **14%**	15 ± 2 **30%**	13 ± 1 **26%**

**Table 4:** Loss of oil fractions in relation to control.

Fraction	Removal of Oil Fractions, %			
	Consortium No. 1	Consortium No. 2	Consortium No. 3	Consortium No. 4
Hexane	21	32	37	32
Benzene	22	22	56	22

**Table 6:** Changes in microbial number in soil in a microfield experiment.

Date	Control		Biopreparation	
	Total Microbial Number, CFU/g Soil	Degrader Number, CFU/g Soil	Total Microbial Number, CFU/g Soil	Degrader Number, CFU/g Soil
30.05	7.2 × 10^6^	1.0 × 10^5^	1.2 × 10^8^	9.6 × 10^7^
14.06	4.0 × 10^6^	1.0 × 10^5^	4.7 × 10^7^	8.5 × 10^6^
29.06	3.2 × 10^6^	4.5 × 10^5^	1.4 × 10^8^	5.0 × 10^7^
17.07	3.6 × 10^6^	4.0 × 10^5^	2.0 × 10^7^	1.4 × 10^6^

## Data Availability

Data available from public libraries/open sources according to references.

## Supplementary Materials

The following supporting information can be downloaded at: https://www.mdpi.com/article/10.3390/microorganisms11020522/s1. Figure S1: Chromatogram of residual n-alkanes after oil biodegradation by consortium No. 3 (red) compared to control (black); Figure S2: Chromatogram of residual polyaromatic hydrocarbons after oil biodegradation by consortia No. 3 (blue) and No 4 (red); Table S1: Data from gravimetric analysis of the degree of oil degradation in the samples analyzed; Table S2: N-alkane content in oil samples before and after culturing of microbial consortia No. 3 and No. 4 in liquid Evans mineral medium with 15% oil for 50 days at 24 °C; and Table S3: PAH content (µg) in oil samples before and after culturing microbial consortia in liquid mineral Evans medium with 15% oil for 50 days at 24 °C.
