# Supplementary figures and images for: Bioremediation of Oil-Contaminated Soil of the Republic of Kazakhstan Using a New Biopreparation
Source: Microorganisms. 2023 Feb 18;11(2):522. doi: 10.3390/microorganisms11020522 (PMC9960684; doi:10.3390/microorganisms11020522)

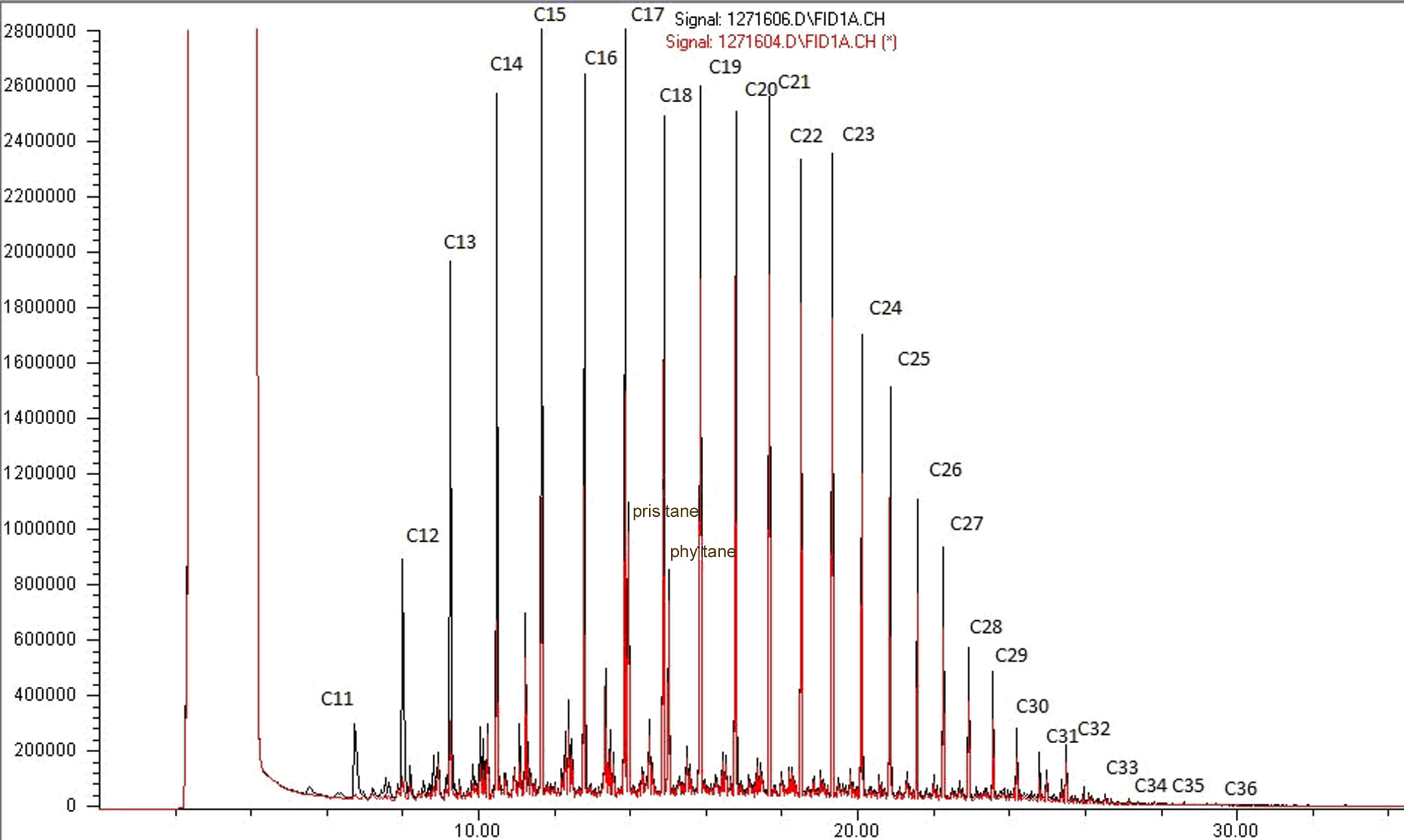

Supplement: Supplementary file 1 [file microorganisms-11-00522-s001.zip › Figure S1.jpg]

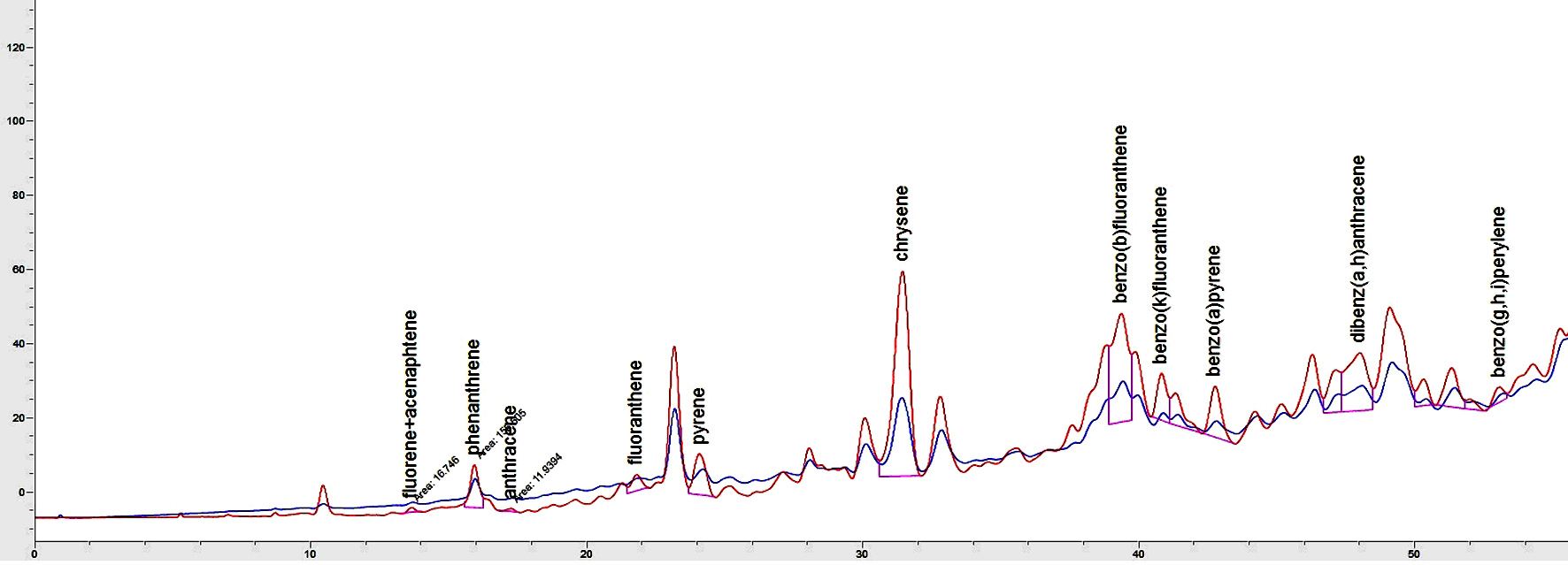

Supplement: Supplementary file 1 [file microorganisms-11-00522-s001.zip › Figure S2.jpg]
