# Supplementary material for: Bioremediation of Oil-Contaminated Soil of the Republic of Kazakhstan Using a New Biopreparation
Source: Microorganisms. 2023 Feb 18;11(2):522. doi: 10.3390/microorganisms11020522 (PMC9960684; doi:10.3390/microorganisms11020522)
Supplement: Supplementary file 1 [file microorganisms-11-00522-s001.zip › Table S1.doc]

Table S1: Data from gravimetric analysis of the degree of oil degradation in the samples analyzed

| Parameter | Control | Abiotic degradation | Consortium No. 1 | Consortium No. 2 | Consortium No. 3 | Consortium No. 4 |
| --- | --- | --- | --- | --- | --- | --- |
| Weight of a dry sample of residual oil, mg | 117±6 | 95±5 | 58±3 | 24±1 | 15±1 | 19±1 |
| Oil degradation, % | 0 | 19 | 50 | 80 | 87 | 84 |

Notes: control – Evans medium + 2% oil; abiotic degradation – Evans medium + 2% oil (cultivation in 10 days at 24°C); ± standard deviation
