# Supplementary material for: Bioremediation of Oil-Contaminated Soil of the Republic of Kazakhstan Using a New Biopreparation
Source: Microorganisms. 2023 Feb 18;11(2):522. doi: 10.3390/microorganisms11020522 (PMC9960684; doi:10.3390/microorganisms11020522)
Supplement: Supplementary file 1 [file microorganisms-11-00522-s001.zip › Table S2.doc]

Table S2. N-alkane content in oil samples before and after culturing of microbial consortia No. 3 and No. 4 in liquid Evans mineral medium with 15% oil for 50 days at 24°C

| alkanes | control | No. 3 | No. 4 |
| --- | --- | --- | --- |
|  | mg | | |
| С_11_H_24_ | 47,5 | 3,4 | 12,9 |
| С_12_H_26_ | 89,9 | 7,2 | 26,5 |
| С_13_H_28_ | 127,8 | 18,1 | 47,2 |
| С_14_H_30_ | 136,1 | 32,8 | 65,7 |
| С_15_H_32_ | 161,7 | 59,4 | 98,8 |
| С_16_H_34_ | 141,2 | 51,7 | 91,5 |
| С_17_H36 | 148,7 | 68,8 | 106,8 |
| prystane | 57,1 | 50,6 | 56,2 |
| С_18_H_38_ | 132,4 | 76,8 | 107,3 |
| phytane | 45,8 | 40,5 | 45,1 |
| С_19_H_40_ | 144,2 | 96,2 | 123,3 |
| С_20_H_42_ | 135,7 | 94,3 | 117,2 |
| С_21_H_44_ | 137,1 | 98,1 | 120,1 |
| С_22_H_46_ | 115,6 | 84,2 | 102,6 |
| С_23_H_48_ | 115,7 | 83,9 | 103,0 |
| С_24_H_50_ | 81,5 | 57,9 | 72,8 |
| С_25_H_52_ | 73,5 | 50,5 | 65,1 |
| С_26_H_54_ | 51,7 | 35,4 | 45,7 |
| С_27_H_56_ | 43,6 | 29,1 | 38,0 |
| С_28_H_58_ | 24,3 | 15,4 | 21,0 |
| С_29_H_60_ | 22,8 | 14,0 | 19,1 |
| С_30_H_62_ | 12,5 | 7,3 | 10,3 |
| С_31_H_64_ | 8,3 | 5,2 | 6,8 |
| С_32_H_66_ | 4,3 | 2,4 | 2,8 |
| С_33_H_68_ | 0,2 | 0,1 | 0,2 |
| С_34_H_70_ | 1,5 | 0,8 | 1,1 |
| С_35_H_72_ | 0,3 | 0,6 | 0,3 |
| С_36_H_74_ | 0,7 | 0,4 | 0,5 |
| **alkanes total** | 2061,5 | 1080,5 | 1507,7 |
| **degradation of alkanes** | **%** | | |
|  |  | **48** | **27** |
