# Supplementary material for: Bioremediation of Oil-Contaminated Soil of the Republic of Kazakhstan Using a New Biopreparation
Source: Microorganisms. 2023 Feb 18;11(2):522. doi: 10.3390/microorganisms11020522 (PMC9960684; doi:10.3390/microorganisms11020522)
Supplement: Supplementary file 1 [file microorganisms-11-00522-s001.zip › Table S3.doc]

Table S3. PAH content (µg) in oil samples before and after culturing microbial consortia in liquid mineral Evans medium with 15% oil for 50 days at 24°C

| PAH | control | No. 3 | No. 4 | No. 3 | No. 4 |
| --- | --- | --- | --- | --- | --- |
|  | μg | | | degradation, % | |
| fluorene | 180 | 90 | 120,6 | 50 | 33 |
| acenaphthene | 205,2 | 108 | 136,8 | 47 | 33 |
| phenanthrene | 876,2 | 576 | 709,2 | 34 | 19 |
| anthracene | 176,4 | 77,4 | 115,2 | 56 | 35 |
| fluoranthene | 194,0 | 154,8 | 177,8 | 20 | 8 |
| pyrene | 127,1 | 57,6 | 60,5 | 55 | 52 |
| benzo(a)anthracene | 0 | 0 | 0 | 0 | 0 |
| chrysene | 135 | 77,4 | 95,8 | 43 | 29 |
| benzo(b)fluoranthene | 496,1 | 312,5 | 414 | 37 | 17 |
| benzo(k)fluoranthene | 24,0 | 9,0 | 12,6 | 62 | 48 |
| benzo(а)pyrene | 21,2 | 6,8 | 8,9 | 68 | 58 |
| dibenzo(a,h)anthracene | 66,6 | 59,4 | 57,6 | 11 | 14 |
| benzo(g,h,i)perylene | 24,1 | 15,4 | 22,0 | 36 | 9 |
| total PAH | 2526,0 | 1544,3 | 1930,9 |  |  |
| **PAH degradation (total), %** |  |  |  | **39** | **24** |
